# Editorial: New trends in emotional intelligence: conceptualization, understanding, and assessment

**DOI:** 10.3389/fpsyg.2023.1266076

**Published:** 2023-08-21

**Authors:** Marina Fiori, Sergio Agnoli, Sarah K. Davis

**Affiliations:** ^1^Research and Development, Swiss Federal University for Vocational Education and Training, Renens, Switzerland; ^2^Department of Life Sciences, University of Trieste, Trieste, Italy; ^3^School of Psychology, University of Worcester, Worcester, United Kingdom

**Keywords:** attitudes, emotional intelligence, emotion information processing, meta-emotion, trauma

## Overview

Over the years, significant strides have been made in refining the conceptualization and measurement of Emotional Intelligence (EI) (e.g., Brackett and Mayer, 2003; Joseph and Newman, 2010; Andrei et al., 2016; O'Connor et al., 2019). Researchers have developed various tools and methods to assess both ability EI and trait EI, providing valuable insights into individuals' emotional capabilities and tendencies. These advancements have helped distinguish different aspects of EI, understand its underlying mechanisms, and identify real-world implications (Martins et al., 2010; Perera and DiGiacomo, 2013; Miao et al., 2017; MacCann et al., 2020). However, the field of EI is ever evolving, and new perspectives continue to emerge. The current Research Topic identifies emerging trends in EI research that draw from various approaches. More specifically, the work of D'Amico and Geraci and Maddocks addresses the conceptualization of EI, Gottfredson and Becker examine factors that may influence emotionally intelligent skills, and Gillioz et al. explore the assessment of EI.

### Conceptualizing EI

D'Amico and Geraci introduce the concept of Meta-emotional intelligence (MEI) which is a multidimensional construct comprising the cognitive aspects of emotional abilities and meta-emotional dimensions, such as beliefs about emotions, self-concept regarding emotional abilities, and self-evaluation of performance. MEI is defined by three key metacognitive processes: meta-emotional knowledge (awareness of emotional abilities), metacognitive self-evaluation (ability to assess one's emotional performance accurately), and meta-emotional beliefs (beliefs about emotions' nature and controllability). The authors suggest that self-report and performance-based measures of emotional intelligence tap different mental processes and advocate for examining the discrepancies between the two under a metacognitive perspective. They therefore introduce the IE-ACCME test, a multi-method tool developed to measure the various components of MEI. Data is presented to suggest that a “harmonious profile” (similar scores in ability and meta-emotional dimensions) leads to better emotional regulation and decision-making, while discrepancies in this profile can result in poor emotional control and behavioral issues. MEI shows higher plasticity compared to emotional intelligence (EI), making it more amenable to change. The article also discusses the potential applications of MEI in promoting emotional awareness and reducing biases in evaluating emotional abilities in preadolescents and adolescents. Overall, MEI offers a promising and innovative approach to understanding emotional intelligence.

The paper by Maddocks suggests that attitudes, particularly implicit and explicit attitudes, may serve as underlying antecedents for ability-EI, trait EI and mixed methods, which are labeled emotional efficacies (EE). After introducing main issues in ability emotional intelligence (EI) and emotional efficacies (EE), Maddocks proposes an attitude-based approach to understanding their relationship. The field of EI research has seen a shift toward more integrative approaches, with some models suggesting that EE traits and competencies are outcomes of ability-EI, while others propose that they may be antecedents. However, the exact relationship between EI and EE is still not fully established. The paper highlights the importance of attitudes in influencing cognitive and emotional processes. Attitudes are evaluative and can trigger emotional responses, which, in turn, influence thoughts and behaviors. By incorporating attitudes into existing models of EI and EE, a more comprehensive understanding of their interplay can be achieved. The author suggests that attitudes play a vital role as they can be explicit (conscious) or implicit (unconscious) and can significantly influence emotional processing and behavior. The inclusion of implicit and explicit attitudes enhances the understanding of how EI and EE operate both automatically and consciously. The proposed attitude-based approach may have several benefits, including better differentiation between conscious and automated processing of EI/EE, a basis for intrapersonal and interpersonal aspects of EI/EE, ethical considerations, and support for personal development in EI and EE.

### Understanding EI

This narrative review by Gottfredson and Becker explores the connection between psychological trauma and emotional intelligence (EI) based on neuroscience and psychology research. It argues that past psychological trauma can negatively impact brain areas and functions related to EI. Two neural adaptations resulting from trauma are hypervigilance and dissociation, which can impair post-trauma brain network functionality and EI abilities. Hypervigilance leads to overactive salience network, reducing positive emotions and hindering emotional recognition and regulation. Dissociation, on the other hand, results in the suppression of salience network, leading to decreased emotional self-awareness and reduced ability to recognize and respond to others' emotions. The review summarizes research which suggests that healing in brain areas and network functionality involved in EI is possible, even after neurological effects of trauma. Recognizing the impact of psychological trauma on EI opens the door for understanding variations in EI and exploring ways to improve EI through recovery processes.

### Assessing EI

The study by Gillioz et al. provides new insights into emotional information processing and its impact on emotional intelligence. In recent studies (e.g., Fiori et al., 2022), Emotion Information Processing (EI_P_) has emerged as a new component of emotional intelligence (EI). More specifically, it has been proposed that EI is not a singular construct, but comprises two distinct components: (1) Emotion Knowledge Component (EI_K_) and (2) Emotion Processing Component (EI_P_). EI_K_ refers to higher-order reasoning about emotions and is typically measured by performance-based EI tests, assessing knowledge about emotions. On the other hand, EI_P_ involves bottom-up processing of emotions and evaluates more spontaneous and rapid emotion information processing. To explore the EI_P_ component, the authors developed a task aimed at measuring fine-grained discrimination of emotional expressions using morphed faces with blended emotions and administered this to 154 participants, alongside measures of emotion recognition, understanding, management, and general intelligence. The results indicated that all facets of EI independently predicted accuracy in the discrimination task, with emotion recognition being the strongest predictor. After controlling for emotion recognition, emotion understanding still predicted accuracy for less difficult stimuli. These findings support the idea that individuals with higher EI possess superior emotion processing skills, particularly during the emotion perception stage of information processing. The task used in the study appears to measure more spontaneous processing of emotional expressions, different from traditional ability EI tests. It includes complex emotional expressions and requires quick responses, making it suitable for assessing individual differences in EI_P_.

## Conclusion

We hope that these papers advance the reader's understanding of EI and its measurement and will inspire continued innovation in measurement approaches, leading to further insights into the nature of EI and its role in various domains, such as education, workplace performance, and mental health.

## Author contributions

MF: Conceptualization, Writing—original draft. SA: Writing—review and editing. SD: Writing—review and editing.
